# Two-Way Social Media Messaging in Postoperative Cataract Surgical Patients: Prospective Interventional Study

**DOI:** 10.2196/jmir.8330

**Published:** 2017-12-19

**Authors:** Thuss Sanguansak, Katharine E Morley, Michael G Morley, Kavin Thinkhamrop, Jaruwan Thuanman, Isha Agarwal

**Affiliations:** ^1^ Department of Ophthalmology Faculty of Medicine Khon Kaen University Khon Kaen Thailand; ^2^ Department of Medicine Massachusetts General Hospital Boston, MA United States; ^3^ Harvard Medical School Boston, MA United States; ^4^ Ophthalmic Consultants of Boston Department of Ophthalmology Harvard Medical School Boston, MA United States; ^5^ Data Management and Statistical Analysis Center Faculty of Public Health Khon Kaen University Khon Kaen Thailand; ^6^ Harvard School of Public Health Boston, MA United States

**Keywords:** cataract, social media, postoperative care, medication adherence

## Abstract

**Background:**

Social media offers a new way to provide education, reminders, and support for patients with a variety of health conditions. Most of these interventions use one-way, provider-patient communication. Incorporating social media tools to improve postoperative (postop) education and follow-up care has only been used in limited situations.

**Objective:**

The aim of this study was to determine the feasibility and efficacy of two-way social media messaging to deliver reminders and educational information about postop care to cataract patients.

**Methods:**

A total of 98 patients undergoing their first eye cataract surgery were divided into two groups: a no message group receiving usual pre- and postop care and a message group receiving usual care plus messages in a mobile social media format with standardized content and timing. Each patient in the message group received nine messages about hand and face hygiene, medication and postop visit adherence, and links to patient education videos about postop care. Patients could respond to messages as desired. Main outcome measures included medication adherence, postop visit adherence, clinical outcomes, and patients’ subjective assessments of two-way messaging. The number, types, content, and timing of responses by patients to messages were recorded.

**Results:**

Medication adherence was better in the message group at postop day 7, with high adherence in 47 patients (96%, 47/49) versus 36 patients (73%, 36/49) in the no message group (*P*=.004), but no statistically significant differences in medication adherence between the groups were noted at preop and postop day 30. Visit adherence was higher at postop day 30 in the message group (100%, 49/49) versus the no message group (88%, 43/49; *P*=.03) but was 100% (49/49) in both groups at postop day 1 and 7. Final visual outcomes were similar between groups. A total of 441 standardized messages were sent to the message group. Out of 270 responses generated, 188 (70%) were simple acknowledgments or “thank you,” and 82 (30%) responses were questions that were divided into three general categories: administrative, postop care, and clinical issues. Out of the 82 question responses, 31 (11%) were about administrative issues, 28 (10%) about postop care, and 23 (9%) about clinical symptoms. All the messages about symptoms were triaged by nurses or ophthalmologists and only required reassurance or information. Patients expressed satisfaction with messaging.

**Conclusions:**

Two-way social media messaging to deliver postop information to cataract patients is feasible and improves early medication compliance. Further design improvements can streamline work flow to optimize efficiency and patient satisfaction.

## Introduction

### Background and Rationale

Social media platforms on mobile phones have changed the way the world communicates. Medicine as a profession is beginning to embrace these new communication modalities to improve health care delivery. This opportunity is valuable in both high income countries as a means for improving patient engagement and education and in low- and middle-income countries where the limitations of overburdened clinics and staff, transportation barriers, and health literacy issues are challenges to providing health care.

Many mobile health (mHealth) interventions have been studied, including apps to enhance patient education, promote behavior change, and improve medication and visit adherence. Others are directed to improving health care delivery systems through electronic decision support, data sensors, data collection, patient tracking, and electronic health records [1]. There is a growing body of research supporting the use of texting to improve patient follow-up [2-4] and medication adherence in chronic diseases such as HIV, tuberculosis, and hypertension [5-10]. Surgical specialties are starting to utilize mHealth technology to improve postop care. Armstrong et al [11] showed a significant decrease in in-person follow-up visits and higher patient convenience scores after breast reconstructive surgery in patients using a mobile app for follow-up using photos and responses to validated recovery and pain surveys. Semple et al [12] also used photos and validated quality of recovery questionnaires for postop follow-up of breast reconstruction and orthopedic surgical patients demonstrating feasibility and acceptability. In both of these studies, surgeons monitored questionnaires and photos and contacted patients based on this information. Another study in China used a social messaging app (WeChat) for follow-up of head and neck surgical patients in China that was shown to decrease patients lost to follow-up, improve patient satisfaction, save physicians’ time, and be more cost-effective compared with usual care [13]. This study allowed for patient-initiated contact through the messaging app for questions.

mHealth interventions to enhance patient adherence have been used in ophthalmology. Some specific examples include medication adherence in glaucoma [14], general outpatient clinic visit adherence [15,16], and postsurgical follow-up for pediatric cataract [17] and trabeculoplasty [18]. Cataract surgery has not yet been impacted by social media and messaging apps. This may be related to the lower use of these platforms in elderly populations with vision problems, but that is starting to change as patients, even older ones, increasingly use and rely on this technology [19]. In low- and middle-income countries, improving outcomes for cataract surgery requires better patient education, compliance with medications, postop follow-up, and outcome monitoring [20,21]. mHealth technology and use of social media platforms are potential ways to help address these gaps.

### Specific Aim

The specific aim of this research project was to determine the feasibility and efficacy of two-way social media messaging in postop cataract surgery patients. We hypothesize that redesigning patient communication and education regarding cataract surgery postop care using a mobile messaging app is feasible and can positively impact the quality and patient experience of postop cataract surgical care.

## Methods

### Setting

Patients were recruited from the ophthalmology clinic at Srinagarind Hospital, the academic teaching hospital for the Faculty of Medicine at Khon Kaen University and the main tertiary referral center for northeast Thailand. Khon Kaen is the largest city in northeast Thailand with a growing urban population and economy. The surrounding provinces, from which most patients come, are rural, agricultural-based, and less developed. Smartphones are widely available in Khon Kaen and surrounding provinces, with mobile apps being a primary mode of communication. In Thailand, SMS text messaging (short message service, SMS) is relatively expensive and not commonly used; messaging apps such as LINE are the preferred mode of communication.

### Study Design

This is an interventional study with two groups. The control group received standard pre- and postcataract surgical care and education and included watching four educational videos on pre- and postop care in the clinic. The intervention group received standard pre- and postcataract surgical care and education, plus nine messages with standardized content and delivery schedule using the LINE messaging app (Multimedia Appendix 1). Message content included information about hand and face hygiene, medication instillation and adherence, visit reminders and adherence, medication reminders, and medication tapering schedule. Patients received a link to the same clinic educational videos on YouTube describing the preop and postop eye care and use of drops so they could watch them on demand (Multimedia Appendices 2-5).

Measurements of medication adherence, visit compliance, and clinical outcomes were obtained at the preop visit, postop day 7, and postop day 30 and compared between the two groups. The other study outcomes focused on the implementation and user experience of the LINE message group and included the number, type, content, and timing of the patient responses to messages. Clinical outcomes were measured, including visual acuity, intraocular pressure, and intraocular inflammation. Patients in the message group also completed a survey regarding subjective assessment of the message content and frequency (Multimedia Appendix 6).

The LINE social media messaging app was selected for this study because it is the most widely used mobile messaging app in the region, is free, has end-to-end encryption for one-to-one chats, automatic deletion from the server after set time, privacy settings to prevent sharing, allows for video links, and the LINE chat history could be downloaded for analysis after the study period.

### Participant Recruitment

Patients recommended to have cataract surgery at the outpatient ophthalmology department at Srinagarind Hospital, Khon Kaen University who met the eligibility criteria were recruited for the study. Recruitment of patients was done in a before and after fashion, recruiting 49 patients for the nonintervention group first, followed by 49 patients in the intervention. This form of patient recruitment was employed to minimize the patient groups from comingling and discussing study-related variables, clinical care, or messages. Patients were eligible for participation in the study if they (1) did not have previous cataract or other eye surgery, (2) had access (directly or through surrogate) to a smartphone and successfully demonstrated facility using the LINE app to send or receive messages and view videos, (3) agreed to follow security settings for LINE study communication, (4) agreed to maintain mobile phone or data service or Wi-Fi service, and (5) agreed to notify the study coordinator if their mobile phone number changed. Patients were excluded if they could not fulfill the above criteria, were younger than 18 years, or were from vulnerable populations including prisoners, those with mental illness, and pregnant women.

Patients in the intervention group could elect to have the messages sent to themselves or a designated surrogate who took responsibility to inform the patient of the messages. Surrogates were direct family members (usually son or daughter) living in the same house. The reasons for using a surrogate typically related to older patients’ lack of familiarity with smartphone usage or visual impairment from cataract preventing screen visualization.

### Study Variables

The study variables collected in the nonmessage and message groups included smartphone user (patient or surrogate), medication adherence scores, visit adherence, and clinical outcomes. Additional information collected from the message group included the number of responses by each patient to each message, types of responses to messages, types of questions sent, timing of responses, and patient feedback survey scores.

Medication adherence was measured with an officially validated Thai translation of the 8-item Morisky Medication Adherence Scale (MMAS-8) [22]. Thai translation was performed by Mapi, an independent linguistic institution that provides validated translations of the MMAS-8, and reviewed by linguists at Khon Kaen University. Similar versions have been used in Thailand in studies of medication adherence in diabetes, HIV, and renal disease [23-25]. MMAS-8 is a widely used tool in the United States and other countries to evaluate medication adherence [26]. Patients in the intervention and control groups completed the MMAS-8 at the preop visit to assess their baseline medication adherence behavior and then at POD-7 (postop day 7) and at POD-30 (postop day 30) visits. Patients were given a score of low, medium, or high medication adherence in accordance with the MMAS-8 scoring criteria (low <6, medium 6<8, and high=8).

Visit adherence was measured as the percent of patients attending their scheduled postop appointment. Patients who did not keep their scheduled appointment were considered noncompliant. These patients were contacted and asked to return for their follow-up exam and final data collection. Patients who could not be reached or did not return after being contacted were considered “no show.”

Clinical outcomes measured included best corrected (with refraction) visual acuity measured on a Snellen chart at 6 m, intraocular pressure measured with Goldmann tonometry, and intraocular inflammation graded by standardized uveitis nomenclature based on the number of cells per 1 mm^2^ high power field [27].

Responses were categorized into two types: (1) simple acknowledgments (eg, thank you) and questions. The type of media used for response was divided into three groups: sticker or emoji, photo, or text. Questions were subdivided into three types: administrative (eg, appointment or scheduling issue); postop care (eg, medication, dressing, and activity issues); and clinical symptoms requiring triage (eg, discomfort or a change in vision).

At the POD-30 visit, patients in the message group completed a 3-question feedback questionnaire evaluating their impressions regarding the frequency of messages, the content of messages, and recommendation for use of messages to other postop cataract patients.

### Statistical Analysis

The demographic characteristics of the patients were summarized as the mean and standard deviation (SD) for continuous variables and as frequency counts with percentages for categorical variables. MMAS-8 scores for low and medium adherence were combined to perform statistical analysis because of low frequency of responses in some categories. The percentage of MMAS-8 low or medium adherence scores was compared with high adherence scores using chi-square or Fishers exact test, depending on response frequency. The association of MMAS-8 score between message and no message groups were presented as odds ratio (OR) and 95% CI and was obtained using multiple logistic regression and generalize estimating equation (GEE). The adjusted OR were obtained in the same manner with the inclusion of gender, age, and LINE user type into the multivariate model. A *P* value of less than .05 was considered significant. All analyses were performed using Stata (StataCorp) version 13.

### Ethical Issues

Approval was obtained from the Khon Kaen University Ethics Committee in Human Research. Informed consent was obtained from all study participants. The research adhered to the tenets of the Declaration of Helsinki. The research was compliant with the Health Insurance Portability and Accountability Act.

## Results

### Demographics

Table 1 describes the demographics of the study participants. The average age in the message group was 64 versus 68 years in the no message group. Surrogate users of the LINE app was 55% in the message group and 75% in the no message group.

### Medication Adherence

Table 2 shows data regarding MMAS-8 medication adherence scores at preop, POD-7, and POD-30 in the message and no message groups. Medication adherence was better in the message group at POD-7 (96% vs 73%, *P*=.004), but no statistically significant differences in medication adherence between the groups were noted at preop and POD-30. There were 4 patients from the no message group who did not keep their POD-30 appointment and did not reschedule, so MMAS-8 survey could not be completed. There were no differences in MMAS-8 scores between patient and surrogate smartphone users at preop, POD-7, or POD-30 visits (Table 3). The association of MMAS-8 score by message or no message groups at POD-7 adjusted for baseline score, gender, age, and LINE user type (patient vs surrogate) was significant (adjusted OR=8.6; 95% CI 1.7-43.2; *P*=.009), but the overall association of MMAS-8 score between two groups using GEE become nonsignificant (adjusted OR=2.48; 95% CI 0.86-7.18; *P*=.09; Multimedia Appendix 7).

**Table 1:** Demographic data and baseline characteristics of study population.

Study population characteristics		Message, n (%)	No message, n (%)	Overall, n (%)
Number of patients		49 (50)	49 (50)	98 (100)
**Gender**				
	Female	25 (51)	20 (41)	45 (46)
	Male	24 (49)	29 (59)	53 (54)
**Age groups (years)**				
	Less than 65	28 (57)	20 (41)	48 (49)
	65 and above	21(43)	29 (59)	50 (51)
**Line user**					
	Patient	22 (45)	12 (25)	34 (35)
	Surrogate	27 (55)	37 (75)	64 (65)

**Table 2:** Comparison of the 8-item Morisky Medication Adherence Scale (MMAS-8) score in message and no message groups by visit.

Visit		MMAS-8^a^ score (N=98)				*P* value^b^
		Low, n (%)	Medium, n (%)	Low and medium, n (%)	High, n (%)	
**Preop**						
	Message	18 (36)	21 (42)	39 (80)	10 (20)	.8^c^
	No message	22 (44)	18 (37)	40 (82)	9 (18)	
**POD^d^-7**						
	Message	1 (2)	1 (2)	2 (4)	47 (96)	.004^e^
	No message	0 (0)	13 (26)	13 (27)	36 (73)	
**POD-30**						
	Message	0 (0)	6 (12)	6 (12)	43 (88)	.74^e^
	No message	0 (0)	4 (9)	4 (9)	41 (91)	

^a^MMAS-8: 8-item Morisky Medication Adherence Scale.

^b^*P* value reflects low and medium compared with high.

^c^*P* value based on chi-square test.

^d^POD: postop day.

^e^*P* value based on Fishers exact test.

**Table 3:** Comparison of the 8-item Morisky Medication Adherence Scale (MMAS-8) score by a LINE user.

Visit		MMAS-8^a^ score (N=98)				*P* value^b^
		Low, n (%)	Medium, n (%)	Low and medium, n (%)	High, n (%)	
**Preop**						
	Patient	17 (50)	13 (38)	30 (88)	4 (12)	.19^c^
	Surrogate	23 (36)	26 (41)	49 (77)	15 (23)	
**POD^d^-7**						
	Patient	0 (0)	4 (12)	4 (12)	30 (88)	.57^c^
	Surrogate	1 (2)	10 (16)	11 (17)	53 (83)	
**POD-30**						
	Patient	0 (0)	5 (15)	5 (15)	29 (85)	.34^f^
	Surrogate^e^	0 (0)	5 (8)	5 (8)	55 (92)	

^a^MMAS-8: 8-item Morisky Medication Adherence Scale.

^b^*P* value reflects low and medium compared with high.

^c^*P* value based on Fishers exact test.

^d^POD: postop day.

^e^Four patients in surrogate group did not keep POD-30 follow-up, so MMAS-8 score is not available.

^f^*P* value based on chi-square test.

### Visit Adherence

Results of visit adherence scores in the message and no message groups at the preop, POD-7, and POD-30 appointments are summarized in Table 4. Visit adherence was lower in the no message group at POD-30: (43/49) vs 100% (49/49) in the message group, *P*=.03, but visit adherence was otherwise 100% in each group at each visit time point.

### Clinical Outcomes

Table 5 shows clinical outcomes at the preop visit, POD-7, and POD-30. Visual acuity was better at POD-7 in the message group (LogMAR acuity 0.19 vs 0.36, *P*=.02) but was not statistically different between groups at preop or POD-30 visits. After adjusting for age, gender, and LINE user type, the difference in visual acuity at POD-7 remained significant (Multimedia Appendix 8). Intraocular pressure and intraocular inflammation measurements showed no statistically significant differences between groups at any postop visit.

### Number and Type of Responses to LINE Messages

Each of the 49 patients in the message group received nine standardized messages, resulting in 441 total messages sent. Of the 49 patients, 41 (84%) responded to at least one message and 5 patients responded to every message. Each message elicited a response by approximately 50% of the recipients.

Out of 270 total responses, there were 188 (70%) simple acknowledgments such as “thank you” and 82 (30%) questions. Out of 82 question responses, 31 (38%) were about administrative issues (eg, appointments), 28 (34%) about postop care (eg, medications), and 23 (28%) about a clinical symptom (eg, foreign body sensation). All the questions’ responses about symptoms were triaged by nurses or ophthalmologists and only required patient reassurance or additional information about concerns (Multimedia Appendix 9).

Patients responded to messages using three different media types: stickers or emojis, photos, or typed messages. Out of 270 total responses, 25 (9%) included photographs, usually of a pleasant or happy nonclinical nature. There were 6 (2%, 6/270) patients who sent photos of medication bottles, bandages, or their eye to help explain their question. There were 102 (38%, 102/270) responses using “stickers,” which are colorful, cartoon-like replies similar to emoji’s but larger in size to communicate a variety of emotions and feelings such as thankfulness, appreciation, or happiness in the LINE app. A total of 143 responses (53%, 143/270) were a typed message.

### Patient Feedback Regarding LINE Message Frequency and Content

Out of 49 patients who received messages, all of them completed the feedback survey; 46 (94%) judged the number of messages to be “just right,” and 3 (6%) wanted more messages. None wanted fewer messages. There were 48 (98%, 48/49) patients who found the message content helpful, with 16 (32%, 16/49) rating it “just right,” 20 (40%, 20/49) “helpful,” and 12 (24%, 12/49) “very helpful.” One of 49 patients (2%) thought the message content was slightly helpful.

There were 39 patients (79%, 39/49) who “recommended” and 7 patients (14%, 7/49) who “strongly recommended” the messages for future cataract surgery patients. There were 3 patients (6%, 3/49) who were “neutral” about recommending the messages to a cataract patient.

**Table 4:** Visit adherence by message and no message groups at preop, POD-7, and POD-30 (Fishers exact test).

Visit adherence		Message, n (%)	No message, n (%)	*P* value
**Preop**				-
	No	0 (0)	0 (0)	
	Yes	49 (100)	49 (100)	
**POD^a^-7**				-
	No	0 (0)	0 (0)	
	Yes	49 (100)	49 (100)	
**POD-30**				.03^b^
	No	0 (0)	6^c^ (12)	
	Yes	49 (100)	43 (88)	

^a^POD: postop day.

^b^Fishers exact test.

^c^Six patients did not keep the scheduled POD-30 appointment; 2 patients later rescheduled.

**Table 5:** Comparison of clinical outcomes between message and no message groups by preop, POD-7, and POD-30 visits.

Clinical		Message (n=49) Mean (SD^a^)	No message (n=49) Mean (SD)	Mean difference	95% CI	*P* value
**Visual acuity (LogMAR)**						
	Preop	0.90 (0.66)	0.84 (0.55)	0.06	−0.18 to 0.30	.63
	POD^b^-7	0.19 (0.25)	0.36 (0.43)	0.17	0.03-0.31	.02
	POD-30	0.19 (0.25)	0.36 (0.78)	0.16	−0.07 to 0.40	.16
**Intraocular pressure**						
	Preop	14.65 (3.77)	14.27 (3.79)	0.38	−1.14 to 1.91	.62
	POD-7	11.71 (2.61)	12.31 (3.09)	0.59	−0.56 to 1.74	.31
	POD-30	13.16 (3.42)	12.22 (3.62)	0.94	−0.50 to 2.38	.2
**Intraocular inflammation**						
	Preop	0 (0)	0 (0)	0	0	
	POD-7	0.33 (0.62)	0.38 (0.53)	0.05	−0.19 to 0.28	.68
	POD-30	0 (0)	0 (0)	0	0	

^a^SD: standard deviation.

^b^POD: postop day.

## Discussion

### Principal Findings

#### Medication and Visit Adherence

Our study showed that two-way social media messaging in postop cataract surgical patients was feasible and favorably received by patients. The medication and visit adherence of the postop patients were both generally high and left relatively little room for improvement in the intervention group, and both message and nonmessage groups showed improvement in medication adherence score from their baseline, suggesting that patients are more compliant because of the recent surgery. However, medication adherence measurements in the message group at POD-7 were significantly higher than the nonmessage group, suggesting that messages helped patients remember or understand the importance of taking their medications earlier in the postop process. This is potentially helpful in patients at risk of developing cystoid macular edema (CME). Visit adherence was higher, reaching 100% in the message groups at POD-30. The emphasis on visit adherence in the messages may have helped achieve this and is clinically important as complications such as CME can still develop at this time. Final clinical outcomes were not different between the message and nonmessage groups, but the visual acuity was higher at POD-7. As we did not detect a difference in inflammation levels between the message and no message groups, we suspect that this be because of an unknown confounder or chance.

#### Communication Modalities

Social media smartphone messaging apps allow for a variety of communication modalities, and our patients used many of them. Written text was used most frequently; stickers were nearly as popular. Stickers are a signature feature of the LINE messaging app and are widely used in Thailand. They are increasingly popular features on similar social media apps throughout the world. They offer an element of entertainment and sentiment to users. This is an important consideration as lack of patient engagement has been identified as a key barrier in patient adoption of mHealth technologies [28]. The use of photos of the eye, bandages, or medication bottles to help clarify a question was helpful to staff and patients. Ophthalmologists can sometimes benefit from a mobile phone photo of the eye when triaging remotely patients’ complaints. A mobile phone camera with proper focus and flash illumination can generate a clinically useful image in postop cataract patients [29].

### Study Limitations

Our study has several important limitations. We could not confirm if the messages were received as intended though the rapid response suggested that the messages generally were received. Another weakness is that we did not measure phone calls or unscheduled visits to the clinic in the nonmessage group, which would provide valuable information in assessing the impact of our intervention and changes in workload for the clinic staff. Whereas the patient feedback was generally very positive, we did not design the study to determine the optimal frequency and timing of our messages. As our medication adherence measure was a self-reported survey, we acknowledge that it is subject to social desirability bias. This could result in a higher medication adherence score in patients who are concerned about disappointing their surgeon or other members of their care team. We looked for differences in the message and nonmessage groups for medication adherence and visual acuity with regard to age, gender, and LINE user type using a multivariate analysis (GEE) but found no significant differences regarding number, type, or content of messages. However, we were unable to perform this analysis for visit adherence because visit adherence was 100% in all the message groups.

### Comparison With Prior Work

#### Medication and Visit Adherence

Our study differs from most other medication and visit adherence studies for chronic disease management in that it looked at an mHealth intervention in cataract surgery postop care, which is a short-term, acute, episodic process. Our results support that reminders in this population can also be helpful in improving compliance. This aligns with findings in other ophthalmic reminder studies [13-17] and postop reminder studies [12,16,17].

#### Two-Way Messaging

Two-way messaging has the potential to create voluminous, unwanted responses by patients resulting in increased workload for physicians and staff. Armstrong et al reported that patients using a postop home monitoring app for breast reconstruction surgery sent more emails to clinic staff in the first 30 days [11]. We found clinical symptom questions were similar to those typically seen in routine postop cataract surgery patients and tended to cluster in a small number of patients, similar to the pattern seen in a phone-based system. Many of the questions sent by patients in our study could be easily managed by office and nursing staff, who noted a preference to answering messages on their computer when time permitted rather than receiving phone calls that interrupted workflow throughout the day. Greater convenience and efficiency was also reported when members of an orthopedic care team used a mobile phone messaging app (WhatsApp) for communication [30]. Lyu et al found that using WeChat for clinical follow-up of postop head and neck surgical patients in China is more time-efficient than using telephone follow-up [31]. In the next iteration of the project, we plan to create a simplified “thank you-my eye is doing well” reply to help reduce the number of acknowledgment responses, which would reduce the workload for staff.

#### Future Directions

The research supporting the use of mobile phone apps throughout the perioperative period is increasing in the preoperative, perioperative, and postop periods [32]. Previous research in tele-ophthalmology has demonstrated benefits in screening and remote diagnosis in retinal diseases, glaucoma, pediatric retinopathy, and emergency triage [33]. Our study addresses the opportunity for improving compliance with postop care using social messaging in comparison with some of the other most recent research using mHealth in postop care that focus on self-management tools. These include symptoms surveys, wound photos, and educational information [11,34-36]. Incorporating similar tools in our intervention is an area for further development and research.

To realize the benefits of using social media postop messaging at scale, we see a critical need to develop a robust program to automate messaging for patients at different stages of the postop process and the capacity to adapt these messages for changes in patient management. Other postop studies have incorporated alerts to help automate the monitoring process [11,12], but this does not eliminate the need for managing outliers and adjustments to a patient’s postop care plan. Follow-up with surgeons participating in one postop home monitoring study following questionnaires and photos raised concerns about managing the workload of monitoring patient responses, even without incorporating patient-initiated communications [12].

Another issue requiring further study is to determine the operational impact, including cost and time efficiency of two-way postop messaging. In clinics with a large volume of cataract surgery, this is important information that will ultimately determine uptake of this technology. Low resource settings may not have the option to devote staff to this system unless financial or operational benefits can be demonstrated.

### Conclusions

Using two-way social media messaging for postop care is a promising innovation for not only cataract surgery but other surgical specialties. In addition to improving medication and visit adherence, our impression was that the patients liked the feeling of safety and connection to their care team provided by repeated messaging.

## Appendix

Multimedia Appendix 1 Description of pre- and postop cataract surgical care.

Multimedia Appendix 2 Educational video for patients undergoing cataract surgery: before surgery.

Multimedia Appendix 3 Educational video for patients undergoing cataract surgery: day of surgery.

Multimedia Appendix 4 Educational video for patients undergoing cataract surgery: after surgery.

Multimedia Appendix 5 Educational video for patients undergoing cataract surgery: information for family and caregivers.

Multimedia Appendix 6 Patient feedback questionnaire regarding LINE messages.

Multimedia Appendix 7 Association of visual acuity by message or no message groups adjusted for baseline score, gender, age, and LINE user type.

Multimedia Appendix 8 Message response types and media forms.

Multimedia Appendix 9 Message response types and media forms.

## Abbreviations

CME: cystoid macular edema
GEE: generalized estimating equation
mHealth: mobile health
MMAS-8: 8-item Morisky Medication Adherence Scale
OR: odds ratio
POD: postop day
SD: standard deviation
