# Multimedia Appendix Removal and Editorial Warning Regarding the MMAS Scale (Two-Way Social Media Messaging in Post-Operative Cataract Surgical Patients: A Prospective Interventional Study)

**DOI:** 10.2196/12120

**Published:** 2018-12-20

**Authors:** Thuss Sanguansak, Katharine E Morley, Michael G Morley, Kavin Thinkhamrop, Jaruwan Thuanman, Isha Agarwal

**Affiliations:** ^1^ Department of Ophthalmology Faculty of Medicine Khon Kaen University Khon Kaen Thailand; ^2^ Department of Medicine Massachusetts General Hospital Boston, MA United States; ^3^ Harvard Medical School Boston, MA United States; ^4^ Ophthalmic Consultants of Boston Department of Ophthalmology Harvard Medical School Boston, MA United States; ^5^ Data Management and Statistical Analysis Center Faculty of Public Health Khon Kaen University Khon Kaen Thailand; ^6^ Harvard School of Public Health Boston, MA United States

## Authors' Correction

The authors of “Two-Way Social Media Messaging in Postoperative Cataract Surgical Patients: Prospective Interventional Study” (J Med Internet Res 2017;19(12):e413) advise that in order to address concerns raised by the purported holders of intellectual property interests in the Morisky Medication Adherence Scale (“MMAS”), Multimedia Appendix 7 (Morisky Medication Adherence Scale-8 Item questionnaire for postoperative cataract surgery) should be removed from the article. As a result, Appendix 7 was removed and all subsequent appendices 8-10 were renumbered.

The correction will appear in the online version of the paper on the JMIR website on December 10, 2018, together with the publication of this correction notice. Because this was made after submission to PubMed, Pubmed Central, and other full-text repositories, the corrected article also has been re-submitted to those repositories.

## Editorial Notice

This is the second correction we have to publish due to the actions by Steven Trubow and Donald Morisky from the company MMAS Research LLC, the copyright holder of the instrument. The developers of this scale are known to comb the literature and ask those who used the scale for research to pay for a retroactive license which may cost thousands or tens of thousands of dollars, to add references to their work, or to remove details such as the actual instrument used from publications [1].

While it is certainly the prerogative of copyright holders of research instruments to enforce their rights, the Committee on Publication Ethics (COPE) has recently discussed the ethics of the behavior of certain copyright holders who “hold authors to ransom”, and recommends that affected journals emphasize “the fact that this is not good for the advancement of scientific knowledge or in the public interest” [2]. As open access and open science publisher, JMIR Publications could not agree more and we remind our authors of our policies and preference for public and free availability of research tools, including questionnaires [3], in the interest of reproducability and transparency of research. Given the flurry of legal disputes and correction notices related to MMAS (a third one affecting a JMIR journal is forthcoming), we now actively discourage use of MMAS and other instruments which are not available under a Creative Commons Attribution license, and encourage our authors to use or develop/validate new instruments which can be freely reproduced. 

We are also hereby issuing a special call for papers for short paper instruments or electronic tools licensed under Creative Commons (or available under an Open Source license) that can be used instead of MMAS to measure medication adherence, and will waive the article submission fee for such development and validation papers describing new instruments that can be used as a free alternative to MMAS.
