# Supplementary material for: Two-Way Social Media Messaging in Postoperative Cataract Surgical Patients: Prospective Interventional Study
Source: J Med Internet Res. 2017 Dec 19;19(12):e413. doi: 10.2196/jmir.8330 (PMC5750422; doi:10.2196/jmir.8330)
Supplement: Multimedia Appendix 1 [file jmir_v19i12e413_app1.pdf]

MULTIMEDIA APPENDIX 1: Description of Pre and Post-Op Cataract Surgical Care

**Standard Pre-and Post-Operative Care Protocol**			
Time	Eye Drops	Ophthalmologist exam	Patient education
Initial cataract exam	At discretion of surgeon, typically pre-op antibiotics for 3 days	VA, IOP, Slit lamp/90D exam. Posterior segment	-What to expect on day of surgery (video in clinic)<br>-How to use drops
Day of surgery	None	None	Use of eye drops, post op care re: hygiene, eye safety, face cleanliness
Post-op day 1	Antibiotic[a] 1 drop qid<br>NSAID[b] 1drop qid<br>prednisolone1%[b] 1 drop qid	VA, IOP, Slit lamp, Degree of inflammation	-Basic hygiene<br>-How to take drops
Post-op day 7	NSAID[b] 1 drop bid<br>prednisolone 1%[b] 1 drop qid	VA, IOP, Slit lamp, Degree of inflammation	As per ophthalmologist
Post-op day 30	As per ophthalmologist	VA, IOP, Slit lamp, Degree of inflammation	As per ophthalmologist

[a] Antibiotic: fluoroquinolone or antibiotic of choice by surgeon
[b] NSAID and steroid at discretion of surgeon

**LINE Message Content and Time of Delivery**		
Message	Time	Content
1	Pre-op Day 3	To make your eye surgery a success:<br>Wash your face and hands before surgery. Keeping your face and hands clean reduces chance of infection. Please watch the videos on using eye drops so you are prepared following surgery.<br>Video 1: https://youtu.be/vjfcIb1nj7Y<br>Video 2: https://youtu.be/vjfcIb1nj7Y<br>Video 3: https://youtu.be/vjfcIb1nj7Y<br>Video 4: https://youtu.be/vjfcIb1nj7Y
2	Day 0	Rest quietly in a clean place after surgery. You may take paracetamol if you have some pain in the eye. Do not touch the eye. Leave the patch on the eye.<br>Return for a check of the eye tomorrow.
3	Post-op Day 1	Take each of your eye drops 4 times a day (breakfast, lunch, dinner, and bedtime)<br>Keep hands, face and eye clean. Keep appointment next week. Call if any problems.<br>Congratulations for making it through surgery!
4	Post-op Day 7	Reminder- your eye appointment is tomorrow. Do not miss your appointment- it will help your eye heal better. Continue taking each of your eye drops 4 times a day
5	Post-op Day 8	Your eye is healing well! Continue taking the eye drops with a pink top (prednisolone acetate) 4 times a day. Keep hands and face clean, don't rub or touch the eye.
6	Post-op Day 16	Continue taking the pink cap eye drop (prednisolone acetate) eyedrops 4 times a day. Keep hands and face clean
7	Post-op Day 23	You are making good progress! Continue taking the pink capped eyedrop 4 times a day<br>Keep your face and hands clean, do not touch the eye
8	Post-op Day 29	Please keep your eye appointment tomorrow! We need to check the eye and help it heal.
9	Post-op Day 30	Please stop taking the pink capped eye drop. Take care to protect the eye form any injuries. Sunglasses can help with too much brightness.
