# Supplementary material for: Two-Way Social Media Messaging in Postoperative Cataract Surgical Patients: Prospective Interventional Study
Source: J Med Internet Res. 2017 Dec 19;19(12):e413. doi: 10.2196/jmir.8330 (PMC5750422; doi:10.2196/jmir.8330)
Supplement: Multimedia Appendix 6 [file jmir_v19i12e413_app6.pdf]

MULTIMEDIA APPENDIX 3: Patient Feedback Questionnaire Regarding LINE Messages

**Patient Feedback Questions**					
Question	1	2	3	4	5
1.How do you rate the **number** of LINE messages received?	Too many	Ok, could be less	Just right	Ok, could be more	Not frequent enough
2. How do you rate the **content** of the message?	Not helpful	Slightly helpful	Just right	Helpful	Very helpful
3. If you know someone who will have cataract surgery in the future, would you recommend LINE messages?	No, do not recommend	Recommend with reservations	Does not matter	Yes, recommend	Yes, Strongly recommend
