# Supplementary material for: Two-Way Social Media Messaging in Postoperative Cataract Surgical Patients: Prospective Interventional Study
Source: J Med Internet Res. 2017 Dec 19;19(12):e413. doi: 10.2196/jmir.8330 (PMC5750422; doi:10.2196/jmir.8330)
Supplement: Multimedia Appendix 7 [file jmir_v19i12e413_app7.pdf]

MULTIMEDIA APPENDIX 7: Morisky Medication Adherence Scale-8 Item Questionnaire for Post-operative Cataract Surgery

**Morisky Medication Adherence Scale-8 Item[a]** **(with post-operative cataract surgery as specified condition)**		
You indicated that you are taking eye drops for your post-operative cataract surgery. Individuals have identified several issues regarding their medication-taking behavior and we are interested in your experiences. There is no right or wrong answer. Please answer each question based on your personal experience with your post-operative cataract surgery eye drops.		
	**No=1**	**Yes=0**
1. Do you sometimes forget to take your eye drops for post-operative cataract surgery?		
2. People sometimes miss taking their eye drops for reasons other than forgetting. Thinking over the past two weeks, were there any days when you did not take your eye drops for post-operative cataract surgery?		
3. Have you ever cut back or stopped taking your eye drops without telling your doctor, because you felt worse when you took it?		
4. When you travel or leave home, do you sometimes forget to bring along your eye drops for post-operative cataract surgery?		
5. Did you take your eye drops for post-operative cataract surgery yesterday?		
6. When you feel like your vision is under control, do you sometimes stop taking your eye drops?		
7. Taking eye drops every day is a real inconvenience for some people. Do you ever feel hassled about sticking to your treatment plan after post-operative cataract surgery?		
8. How often do you have difficulty remembering to take all your eye drops? (**Please circle your answer below)** Never/Rarely……………………………………….**4** Once in a while…………………………………… **3** Sometimes…………………………………………..**2** Usually………………………………………………..**1** All the time…………………………………………**0**		

[a]Morisky DE, Ang A, Krousel-Wood M, Ward HJ Predictive validity of a medication adherence measure in an outpatient setting. J Clin Hypertens (Greenwich). 2008 May;10(5):348-54.

Use of the ©MMAS is protected by US copyright laws. Permission for use is required. A license agreement is available from: Donald E. Morisky, ScD, ScM, MSPH, Professor, Department of Community Health Sciences, UCLA Fielding School of Public Health, 650 Charles E. Young Drive South, Los Angeles, CA 90095-1772, dmorisky@ucla.edu.
