# Supplementary material for: Two-Way Social Media Messaging in Postoperative Cataract Surgical Patients: Prospective Interventional Study
Source: J Med Internet Res. 2017 Dec 19;19(12):e413. doi: 10.2196/jmir.8330 (PMC5750422; doi:10.2196/jmir.8330)
Supplement: Multimedia Appendix 8 [file jmir_v19i12e413_app8.pdf]

MULTIMEDIA APPENDIX 9: Association of Visual Acuity by Message/No Message Groups Adjusted for Baseline Score, Gender, Age, and LINE User Type

Mean ± standard deviation of Visual acuity and their differences together with their 95% confidence intervals, between message and no message groups, for each of the 2 measurements post operation at day 7 and day 30

Visual acuity (LogMAR)	Message n=49 Mean±SD	No message n=49 Mean±SD	Mean diff.[a]	Mean diff.[b]	95% CI[c]	P-value
Pre-op	0.90±0.66	0.84±0.55	0.06	0.12	-0.12 to 0.37	0.32
POD-7	0.19±0.25	0.37±0.42	0.18	0.15	0.01 to 0.29	0.04
POD-30	0.19±0.25	0.36±0.78	-0.16	-0.20	-0.43 to 0.03	0.09
Overall			0.17	0.17	0.01 to 0.33	0.04

[a] Crude mean difference using simple linear regression
[b] Mean difference adjusted for baseline measurements, LINE user, gender, and age of patients for each visit using multiple linear regression and for overall using GEE implemented under generalized linear model frameworks.
[c] 95% CI for adjusted mean difference
