# Supplementary material for: Two-Way Social Media Messaging in Postoperative Cataract Surgical Patients: Prospective Interventional Study
Source: J Med Internet Res. 2017 Dec 19;19(12):e413. doi: 10.2196/jmir.8330 (PMC5750422; doi:10.2196/jmir.8330)
Supplement: Multimedia Appendix 9 [file jmir_v19i12e413_app9.pdf]

MULTIMEDIA APPENDIX 10: Message Response Types and Media Forms

	Number (%) of Responses	
	**n**	**(%)**
**Response types N=270**		
Thank you	188	(70)
Question	82	(30)
Total	270	(100)
**Question types N=82**		
Administrative issue	31	(38)
Post-op care	28	(34)
Symptoms	23	(28)
Total	82	(100)
**Response media form N=270**		
Photo	25	(9)
Sticker or emoji	102	(38)
Typed message	143	(53)
**Total**	270	(100)
